# Exploring urban land surface temperature using spatial modelling techniques: a case study of Addis Ababa city, Ethiopia

**DOI:** 10.1038/s41598-024-55121-6

**Published:** 2024-03-15

**Authors:** Seyoum Melese Eshetie

**Affiliations:** Space Science and Geospatial Institute of Ethiopia, Remote Sensing Department, Addis Ababa, Ethiopia

**Keywords:** Climate change, Land surface temperature, Ordinary least square, Geographically weighted regression, Hotspot Analysis, Climate-change impacts, Climate-change mitigation, Climate sciences, Climate change, Climate and Earth system modelling

## Abstract

Urban areas worldwide are experiencing escalating temperatures due to the combined effects of climate change and urbanization, leading to a phenomenon known as urban overheating. Understanding the spatial distribution of land surface temperature (LST) and its driving factors is crucial for mitigation and adaptation of urban overheating. So far, there has been an absence of investigations into spatiotemporal patterns and explanatory factors of LST in the city of Addis Ababa. The study aims to determine the spatial patterns of land surface temperature, analyze how the relationships between LST and its factors vary across space, and compare the effectiveness of using ordinary least squares and geographically weighted regression to model these connections. The findings showed that the spatial patterns of LST show statistically significant hot spot zones in the north-central parts of the study area (Moran’s I = 0.172). The relationship between LST and its explanatory variables were modelled using ordinary least square model and thereby tested if there is spatial dependence in the model using the Koenker (BP) Statistic.The result revealed non-stationarity (p = 0.000) and consequently geographically weighted regression was employed to compare the performance with OLS. The research has revealed that, GWR (R^2^ = 0.57, AIC = 1052.1) is more effective technique than OLS (R^2^ = 0.42, AIC = 2162.0) for studying the relationship LST and the selected explanatory variables. The use of GWR has improved the accuracy of the model by capturing the spatial heterogeneity in the relationship between land surface temperature and its explanatory variables. The relationship between LST and its explanatory variables were modelled using ordinary least square model and thereby tested if there is spatial dependence in the model using the Koenker (BP) Statistic. The result revealed non-stationarity ((p = 0.000) and consequently geographically weighted regression was employed to compare the performance with OLS. The research has revealed that, GWR (R^2^ = 0.57, AIC = 1052.1) is more effective technique than OLS (R^2^ = 0.42, AIC = 2162.0) for studying the relationship LST and the selected explanatory variables. The use of GWR has improved the accuracy of the model by capturing the spatial heterogeneity in the relationship between land surface temperature and its explanatory variables. Consequently, Localized understanding of the spatial patterns and the driving factors of LST has been formulated.

## Introduction

Urban areas worldwide are experiencing escalating temperatures due to the combined effects of climate change and urbanization, leading to a phenomenon known as urban overheating. The rise in temperatures within cities poses significant challenges to both the environment and socio-economic systems. Urban overheating has severe implications for many areas of life, including socioeconomic and environmental issues. Land surface temperature is a key factor that contributes to the urban overheating. Understanding the spatial distribution of land surface temperature (LST) and its driving factors is crucial for mitigation and adaptation of urban overheating. Land surface temperature (LST) refers to the temperature of the Earth's land surface and holds significant importance as an environmental factor that impacts multiple facets of urban ecosystems, such as energy consumption, air quality, and public health. Moreover, LST serves as a valuable indicator of the urban heat island effect, a noteworthy environmental challenge in urban areas^1^. Land surface temperature is a key factor that contributes to the urban heat island effect. As urban areas replace natural land covers with impervious surfaces such as concrete and asphalt, they absorb more heat from the sun and radiate it back into the environment, leading to higher temperatures^2^. Research has shown that there is a clear relationship between land surface temperature and the severity of the urban heat island effect^2–4^. Urban heat island has severe implications for many areas of life, including socioeconomic and environmental issues. Air pollution may increase, daytime temperatures become warmer, and nighttime cooling becomes less effective^5–8^. These alterations lead to discomfort and an increase in human premature mortality rates due to excessive heat. In fact, extreme heat is a primary contributor to the rise in weather-related human mortality^3,5^. The increase in LST in urban areas has been linked to several factors associated with land use/land cover change, vegetation cover, and surface materials such as impervious surfaces, are some of the primary drivers of LST^9–11^.

## Motivation and research problem

Addis Ababa is one of the fastest-growing cities in Africa, which has led to the conversion of green spaces and agricultural lands into urban areas^12–14^. Urbanization and development have led to changes in LST in the city, which can have significant impacts on the urban environment and the health and well-being of urban residents^12–16^. This conversion has resulted in an increase in land surface temperature, which thereby contributed to the increase in urban heat island effect^6–8, 17^. With the growth of urbanization, the understanding of the spatial distribution of LST and its explanatory factors is crucial in mitigating the urban heat island effect. The spatial pattern and explanatory variables of land surface temperature (LST) is a critical area of study, given its impact on the environment, human life^18^. While traditional correlation analysis and multivariate regression have been widely used and established, these statistical techniques typically do not consider spatial dependency and are therefore considered non spatial when analyzing existing studies^18^.

Especially, the spatial pattern and explanatory variables of land surface temperature using ordinary least square (OLS) and geographically weighted regression (GWR) have not been well studied^18^. This lack of research on this topic has significant implications for urban planning, environmental management and climate change adaptation^19,20^. The novelty of this research lies in its comparative analysis of OLS and GWR for modeling LST in Addis Ababa city, Ethiopia. The importance of using ordinarily least squares (OLS) and geographically weighted regression (GWR) to understand variations in land surface temperature has been highlighted in numerous studies^21^ and that spatially explicit modeling plays a significant role in assessing potential impacts of climate change on urban environments. This study aims to fill the research gap by comparing the performance of OLS and GWR in modeling the relationship between LST and its driving factors in Addis Ababa city to determine which method provides a better understanding of the spatial patterns and drivers of LST.

The study aims to achieve the following specific objectives: (a) assessing the distribution of land surface temperature (LST) across Addis Ababa and examining the level of spatial autocorrelation. (b) Investigating whether the spatial relationships between LST and its influencing factors vary across different neighborhoods in Addis Ababa. (c) Comparing the effectiveness of spatial regression and Geographical Weighted Regression (GWR) in modeling the association between LST and its influencing factors.

## Materials and methods

### Study area

**Figure 1:**
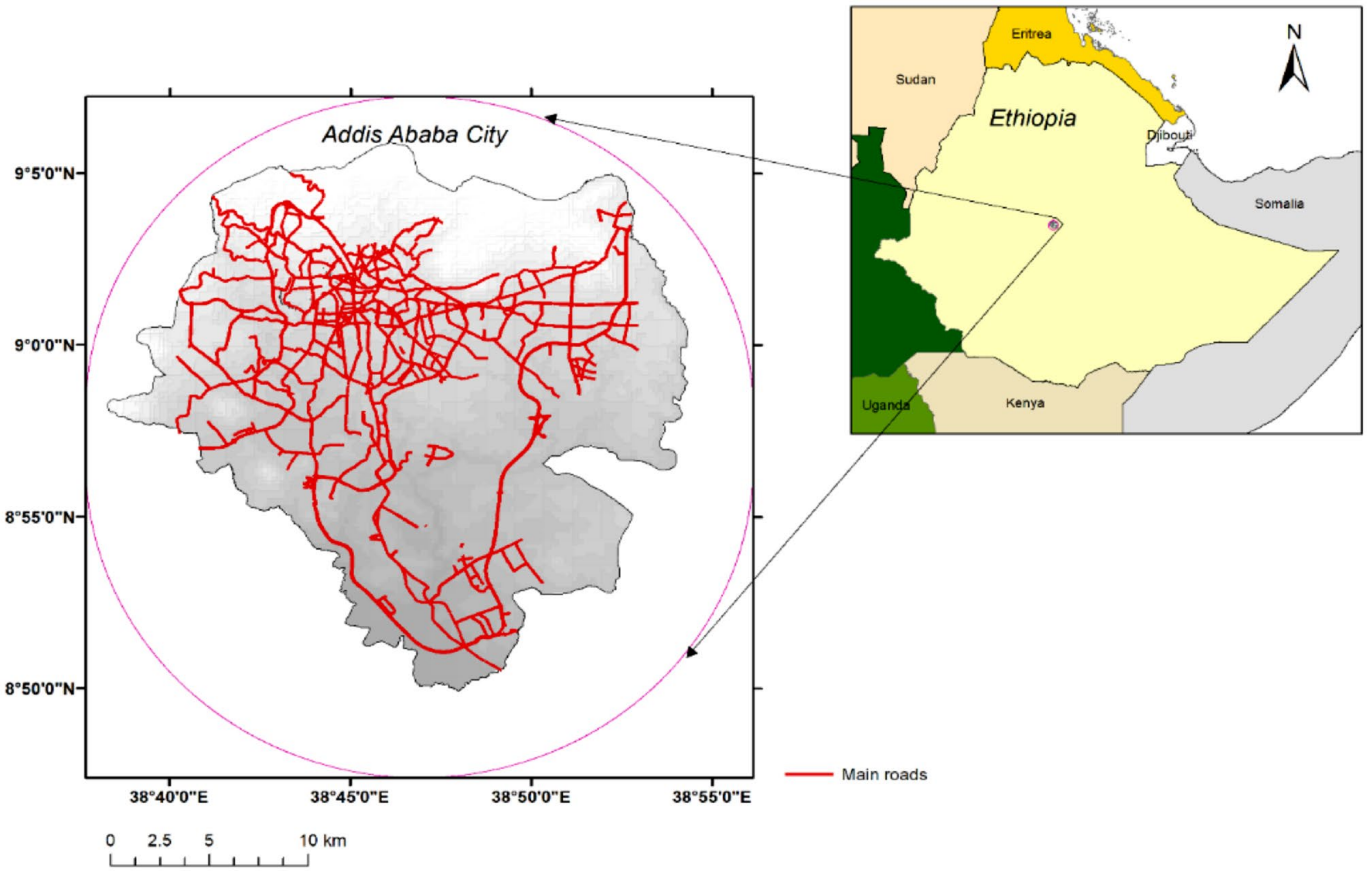
Addis Ababa city as inset map in the Horn of Africa. (Map created by author using QGIS 3.22 software: https://qgis.org/).

Located in the central part of the country, Addis Ababa is the capital and largest city of Ethiopia^15^ (see Fig. 1). The city is located at an elevation of 2400 m above sea level, which gives it a relatively cool and mild climate throughout the year. The average temperature in Addis Ababa is around 16 °C (60 °F).The city is the political, economic, and cultural center of Ethiopia. The city has two main rainy seasons: a long rainy season from June to September, and a short rainy season from February to April. The rainfall during the rainy season is generally moderate, but there can be heavy downpours at times. The rest of the year is relatively dry, with little to no rainfall. Like many other cities in the developing world, it faces numerous challenges related to urbanization, including the urban heat island effect^22^.

### Data source

Landsat data downloaded from the NASA Earth Data portal (https://lpdaac.usgs.gov/) has been used to extract land surface temperature (LST), normalized difference, vegetation index (NDVI), normalized difference built up index (NDBI), and normalized difference water index (NDWI). Elevation data has been also acquired from NASA Earth Data portal (https://lpdaac.usgs.gov/).The climatology of the air temperature and rainfall has obtained from WorldClim (https://www.worldclim.org/).Population density data has been acquired from WorldPop (https://www.worldpop.org/).

### Data preprocessing

#### Extracting land surface temperature (LST) from Landsat data

LST was retrieved from band 10 of the Landsat 8 OLI and TIRS image of Addis Ababa city using the following algorithms^23–25^.

- Calculation of TOA (Top of Atmospheric) spectral radiance
  1 $${\text{TOA }}\left( {\text{L}} \right) \, = {\text{ ML }}*{\text{ Qcal }} + {\text{ AL}}$$

M_L_ = Band-specific multiplicative rescaling factor from the metadata; Q_cal_ = corresponds to band 10.

A_L_ = Band-specific additive rescaling factor from the metadata.

(b) TOA to Brightness Temperature conversion
  2 $${\text{BT}}\frac{k2}{{\ln \left( \frac{k1}{L} \right) + 1}} - {273}.{15}$$

K_1_ = Band-specific thermal conversion constant from the metadata.

K_2_ = Band-specific thermal conversion constant from the metadata.

(c) NDVI Calculation^26^
  3 $${\text{NDVI}} = \frac{{\left( {{\mathbf{NIR}} {-} {\mathbf{R}}} \right) }}{{\left( {{\mathbf{NIR}} + {\mathbf{R}}} \right)}}$$
  where NIR is near infrared band and R is red band.

Subsequently, the proportion of vegetation (P_v_), which is highly related to the NDVI, and emissivity (ε), should be be calculated.

(d) Calculate the proportion of vegetation (P_v)_
  4 $${\text{P}}_{{\text{v}}} = \left[ {\frac{{{\text{NDVI }} - {\text{NDVImin}}}}{{{\text{NDVImax }} - {\text{NDVImin}}}}} \right]^{2}$$
(e) Emissivity (ε)
  5 $$\varepsilon \, = \, 0.00{4 }*{\text{ P}}_{{\text{v}}} + \, 0.{986}$$
(f) Calculate the land surface temperature
  6 $${\text{LST}} = \left[ {\frac{BT}{{(1 + \left( {0.00115{\text{*BT}}/1.4388} \right)}}} \right]*{\text{ln }}(\varepsilon )$$

#### Extracting Normalized Difference Built up Index (NDBI)

The Normalized Difference Built-up Index (NDBI) uses the near infrared (NIR) and shortwave infrared (SWIR) bands to emphasize manufactured built-up areas. It is calculated by taking the difference between the Near-Infrared (NIR) and Short-Wave Infrared (SWIR) bands of satellite imagery, and dividing it by the sum of the same two bands. NDBI is calculated using the algorithm below^26^:

7 $${\text{NDBI }} = \frac{{{ }\left( {{\text{NIR }} - {\text{ SWIR}}} \right){ }}}{{\left( {{\text{NIR }} + {\text{ SWIR}}} \right)}}$$

The values obtained from this calculation range from − 1 to + 1, where positive values represent built-up areas, negative values represent non-built-up areas, and values close to zero represent bare soil or vegetation. NDBI has been found to be effective in distinguishing built-up areas from other land covers. It is widely used in urban studies and land-use planning, as well as in environmental monitoring and disaster management.

#### Extracting Normalized Difference Water Index (NDWI)

Normalize Difference Water Index (NDWI) is use for the water bodies analysis. The index uses Green and Near infra-red bands of remote sensing images. The NDWI can enhance water information efficiently in most cases. NDWI is developed^27^ to enhance the water related features of the landscapes. This index uses the near infrared (NIR) and the Short-Wave infrared (SWIR) bands. NDWI can be calculated by following formula^27^:

8 $${\text{NDWI }} = \frac{{{ }\left( {{\text{NIR }}{-}{\text{ SWIR}}} \right){ }}}{{\left( {{\text{NIR }} + {\text{ SWIR}}} \right)}}$$

### Data analysis

The spatial analysis techniques used in the study includes spatial autocorrelation analysis, spatial interpolation, and hot spot analysis. The study developed maps to visualize the spatial distribution of LST.

#### Spatial autocorrelation

Tobler's First Law of Geography, often referred to as the “law of proximity,” is a fundamental concept in spatial analysis. This law states that “everything is related to everything else, but near things are more related than distant things”^28^ In other words, geographic objects that are closer together are more likely to be connected or interact with each other than objects that are further apart. Spatial autocorrelation measures how much close objects are in comparison with other close objects. Spatial autocorrelation helps understand the degree to which one object is similar to other nearby objects. The presence spatial autocorrelation implies information redundancy and has important implications for spatial data analysis. Moran’s I test is used to quantify spatial autocorrelation. Moran’s I test measure the degree of similarity or dissimilarity between neighboring observations^29^. The Moran’s I Index is a common method used to examine global spatial autocorrelation, which takes into account both the feature locations and attribute values of a dataset. The Moran’s I Index yields values that range from − 1.0 (perfectly dispersed) to + 1.0 (perfectly clustered), making it a useful tool for testing spatial clustering in datasets such as LST. It is based on cross products of the deviations from the mean and is calculated for n observations on variable x at locations *j and I*^29^*.*

9 $${\varvec{I}} = \frac{{\varvec{n}}}{{{\varvec{S}}0}}\user2{ }\frac{{\user2{ }\mathop \sum \nolimits_{{\varvec{i}}} \mathop \sum \nolimits_{{\varvec{j}}} {\varvec{Wij}}({\mathbf{Xi}} - \overline{{{\varvec{X}})}} \user2{ } - ({\mathbf{Xj}} - \overline{{{\varvec{X}})}} }}{{\mathop \sum \nolimits_{{\varvec{i}}} ({\mathbf{Xi}} - \overline{{{\varvec{X}})}} 2}}\;\;\;{\varvec{S}}0 = \mathop \sum \limits_{{\mathbf{i}}} \mathop \sum \limits_{{\mathbf{j}}} {\mathbf{Wij}}$$

Its value range from − 1 to + 1. Moran’s I can be classified as positive, negative and no spatial auto-correlation.

#### Hotspot analysis

Moran’s I index was used to assess whether there were spatial clusters in the LST values dataset. However, it only indicates the presence of spatial clusters or dispersions among the assigned values of a single variable, and does not provide information on the geographical distribution or categories of clusters (such as low value or high value clusters). To address this limitation, the Getis-Ord Gi* statistic is a useful tool for analyzing the spatial distribution of hot spot and cold spot patterns and can provide more detailed information about the categories and geographical patterns of clusters.

Local spatial autocorrelation statistics are observation-specific measures of spatial association^30^. They to detect local spatial clustering around an individual location, they are particularly well suited for finding hot spots. The Getis-Ord Gi* statistics is a local statistic that allows us to discover new locations with significant clusters of hot and cold spots^31^.

10 $${\varvec{G}}_{{\varvec{i}}}^{\user2{*}} = \user2{ }\frac{{\mathop \sum \nolimits_{{{\varvec{i}} = 1}}^{{\varvec{n}}} {\varvec{w}}_{{{\varvec{i}},\user2{j }}} {\varvec{x}}_{{\varvec{j}}} - \overline{\user2{X}}\mathop \sum \nolimits_{{{\varvec{j}} = 1}}^{{\varvec{n}}} {\varvec{w}}_{{{\varvec{i}},{\varvec{j}}}} \user2{ }}}{{{\varvec{s}}\sqrt {\frac{{\left[ {{\varvec{n}}\mathop \sum \nolimits_{{{\varvec{j}} = 1}}^{{\varvec{n}}} {\varvec{w}}^{2}_{{{\varvec{i}},{\varvec{j}}}} \user2{ } - \user2{ }\left( {\mathop \sum \nolimits_{{{\varvec{j}} = 1}}^{{\varvec{n}}} {\varvec{w}}_{{{\varvec{i}},{\varvec{j}}}} } \right)^{2} } \right]}}{{{\varvec{n}} - 1}}} }}$$

where $x_{j}$ the attribute is value for feature *j*; $w_{i,j}$ is the spatial weight between *i* and *j; n* is equal to the total number of features and:

11 $${\overline{\text{X}}} = { }\frac{{\mathop \sum \nolimits_{{{\text{j}} = 1}}^{{\text{n}}} {\text{w}}_{{\text{j}}} }}{{\text{n}}}$$

12 $$\user2{ S} = \user2{ }\sqrt {\frac{{\mathop \sum \nolimits_{{{\varvec{j}} = 1}}^{{\varvec{n}}} {\varvec{x}}^{2}_{{\varvec{j}}} }}{{\varvec{n}}} - \left( {\overline{\user2{X}}} \right)^{2} } \user2{ }$$

#### Spatial regression

Ordinary least square (OLS) is a widely used linear regression method that assumes a constant relationship between the dependent variable (LST) and the independent variables across the study area. GWR, on the other hand, is a spatial regression method that allows for the estimation of regression coefficients at different locations in the study area, taking into account the spatial heterogeneity of the relationship between LST and urban characteristics^32^. The OLS regression equation is calculated using the following formula^32^:

13 $${\varvec{l}} = {\upbeta }_{{0{ } + }} \mathop \sum \limits_{{\text{k}}} {\upbeta }_{{\text{k}}} {\text{X}}_{{{\text{ik}}}} + {\upvarepsilon }_{{\text{i}}} { }$$

${\varvec{l}}$ is the dependent variable for the observation *i.*

***β***_***k***_ is the regression coefficients for the variable *k.*

$\user2{ \beta }_{{0\user2{ }}}$ is the regression intercept*.*

${\varvec{\varepsilon}}_{{\varvec{i}}}$ is the potion of the dependent variable that isn’t explained by the model.

**Figure 2:**
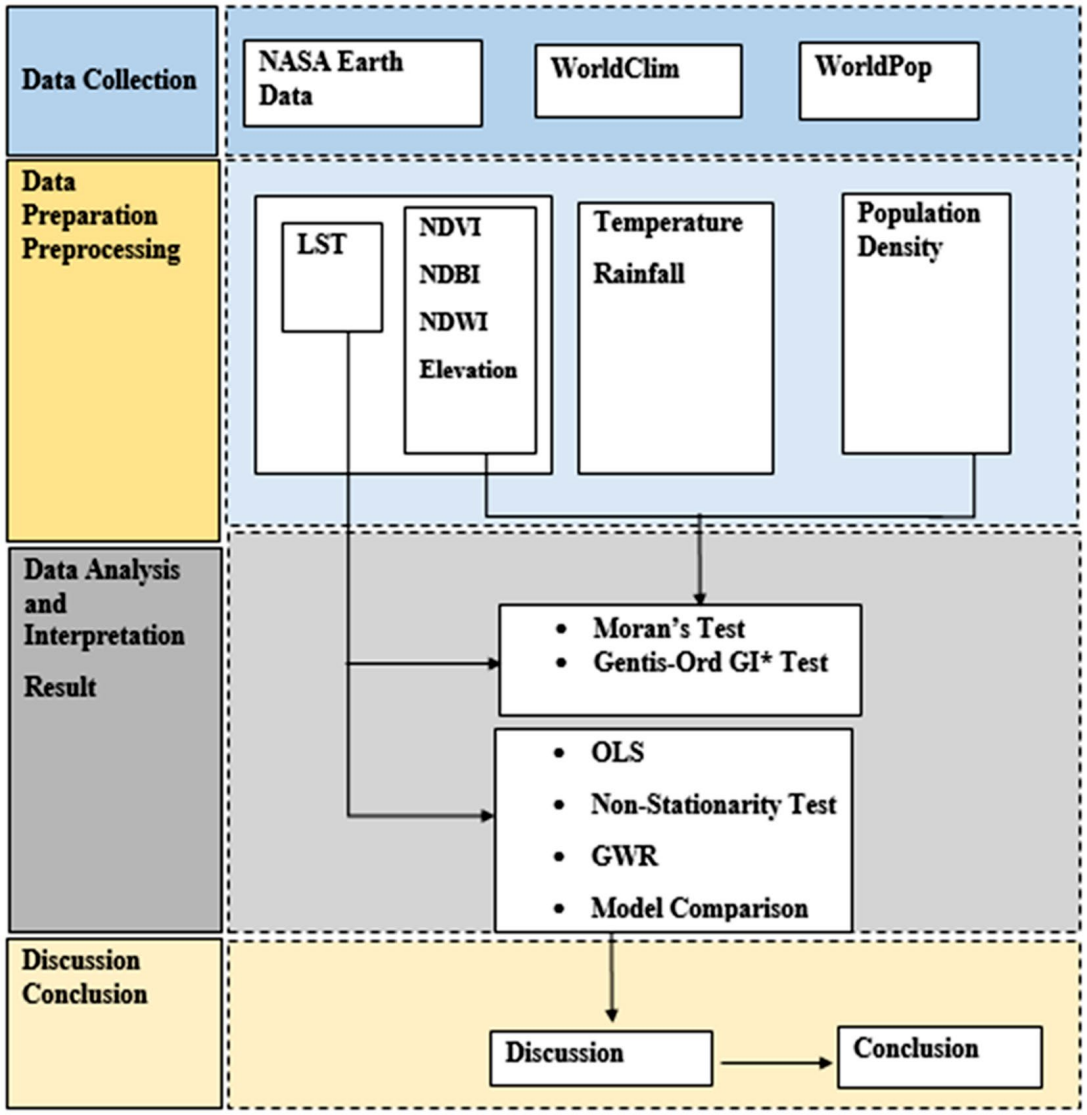
Flowchart of the study method.

The *geographically weighted regression (GWR)* was employed to compare with OLS model. In GWR models, the spatial variability between the response variable (LST) and explanatory is taken into consideration. The GWR is computed using the following formula^32^:

14 $$\gamma = \beta_{0 } \left( {u_{i} ,v_{i} } \right) + \mathop \sum \limits_{k} \beta_{k} \left( {u_{i} , v_{i} } \right)X_{ik} + \varepsilon_{i}$$

where ***u***_***i***_ and ***v***_***i***_ represent the point coordinates of *ith* in space. Thus, the GWR equation (***b***) distinguishes spatial variations in relationships might exist and provides a way in which they can be measured. In spatial regression, the coefficients, standard errors, and t-statistics play important roles in understanding the relationship between the dependent variable and the independent variables. The coefficients represent the estimated effect of each independent variable on the dependent variable. They indicate the change in the dependent variable associated with a one-unit change in the corresponding independent variable, while holding other variables constant. The coefficients provide insights into the direction and magnitude of the relationship between the variables. The standard error measures the accuracy or precision of the coefficient estimates. It quantifies the variability or dispersion of the estimated coefficients around their true values. A smaller standard error indicates a more precise estimate. The t-statistic is a measure of the statistical significance of the estimated coefficients. It assesses whether the coefficient differs significantly from zero, indicating whether the corresponding independent variable has explanatory power in predicting the dependent variable. In summary, the coefficients provide information about the direction and magnitude of the relationship, while the standard errors and t-statistics help determine the statistical significance and precision of the coefficient estimates. The generalized research workflow is presented in Fig. 2.

## Results

### Patterns of land surface temperature

#### Spatial autocorrelation of LST using Moran’s I test

**Figure 3:**
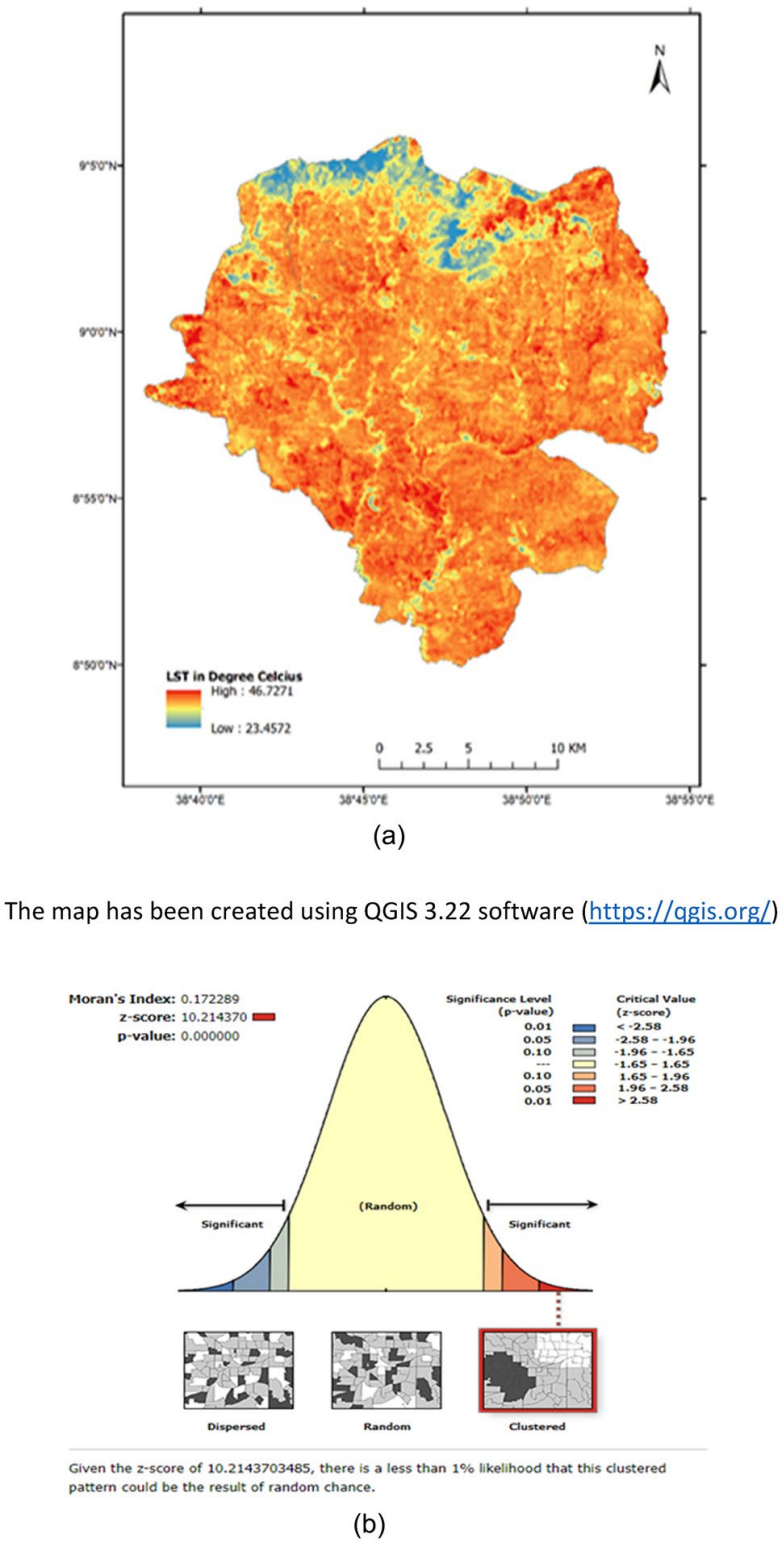
Patterns of land surface temperature. (**a**) Map of LST pattern of Addis Ababa city; (**b**) Moran’s I test of LST. The map has been created using QGIS 3.22 software (https://qgis.org/).

The LST pattern and spatial distribution were extracted from bands 10 and11 of the Landsat 8 Thermal Infrared Sensor (TIRS) image of Addis Ababa city. This process was carried out using QGIS 3.22 software (https://qgis.org/). The results can be visualized in Fig. 3a. Spatial autocorrelation helps identify if there is a systematic spatial variation or clustering in the values of a variable across a geographic area. As depicted in Fig. 3b, the Moran’s I test result of 0.172 suggests that there is clustering of similar LST values within the study area. This clustering could be driven by a range of factors, such as the spatial arrangement of land uses or the distribution of built environment characteristics. To further understand the factors driving the clustering of LST values, it would be useful to conduct additional spatial analyses, such as hot spot analysis or kernel density estimation^33^. These methods is used to identify areas of high or low LST and their spatial patterns in relation to other variables. For example, if high LST values are clustered around areas with low vegetation cover, one could infer that vegetation has a cooling effect on the urban landscape and could be used as a tool to mitigate heat. The Moran’s I test result of 0.172 suggests that there is clustering of LST values in the study area^29^. High levels of spatial autocorrelation in LST indicate that temperature values at nearby locations are more similar to each other than expected by chance. The presence of spatial autocorrelation implies that neighboring locations have similar LST values, which can be attributed to various factors such as land cover, urban morphology, or local climate conditions. Understanding the spatial autocorrelation of LST is important for several reasons. It can provide insights into the underlying processes and drivers of LST patterns help identify areas of high or low LST values, and guide the development of targeted mitigation strategies or urban planning interventions.

The findings showed that the spatial patterns of LST show statistically significant hot spot zones in the north-central parts of the study area (Moran’s I = 0.172).

#### Hotspots of land surface temperature

**Figure 4:**
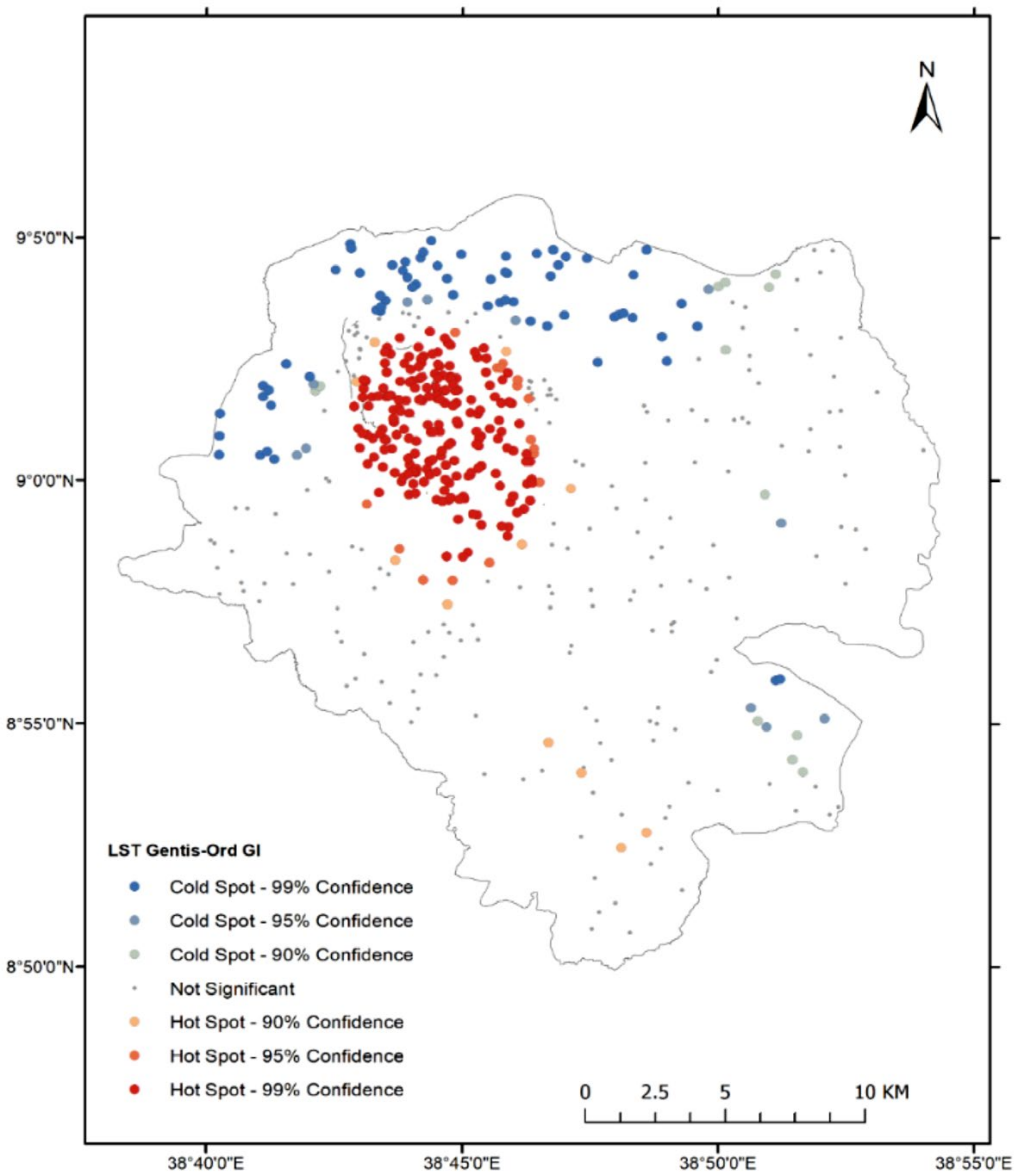
Map of LST hotspot using the Gentis-Ord GI* test. (Map created by author using QGIS 3.22 software: https://qgis.org/).

The Gentis-Ord GI* test is one example of a hotspot analysis technique that is commonly used in geographic information systems (GIS) and spatial statistics^33^. According to the analysis result (see Fig. 4), a hot spot with more than 90% confidence has been discovered in many parts of the study area. The hotspot in LST could indicate specific areas where temperature-mitigating measures are needed, such as increased green space, changes in architectural design or technological interventions. Those areas could be prioritized for urban planning and development initiatives aimed at mitigating the urban heat island effect, which can have both environmental and public health implications.

It is also important to consider other environmental and socio-economic factors that could be contributing to the LST hotspot. For example, certain land use patterns, such as industrial zones or high-density housing, can lead to increased heat. Social vulnerabilities such as low-income or elderly populations could experience the harmful impacts of higher temperatures more intensely. Additional analysis, such as spatial regression models or kernel density estimation, can be used to uncover the causal factors behind the LST hot spot*.*

### Spatial regression

#### Identification of explanatory variables

To identify the most relevant driving factors for land surface temperature (LST), several past research studies were reviewed and possible sources of qualitative evidence were analyzed. From the literature, it was found that various factors influence LST, including air temperature, rainfall, elevation, normalized difference vegetation index (NDVI), normalized built-up index (NDBI), population density, and normalized difference water index (NDWI).Air temperature is a crucial factor that affects LST because it drives the exchange of heat between the atmosphere and the land surface. As air temperature increases, LST tends to increase as well. A study by^5,34^ in Beijing, China found a positive correlation between air temperature *and LST, with LST* increasing by 1.04 °C for every 1 °C increase in air temperature. Normalized Difference Vegetation Index (NDVI) measures the amount and health of vegetation cover in an area^5^. Mean annual rainfall can have significant impacts on LST because it affects the amount of vegetation cover, soil moisture, and evapotranspiration rate^20^. Areas with high mean annual rainfall tend to have more vegetation cover, which can regulate the amount of solar radiation absorbed by the land surface and reduce LST. Additionally, higher soil moisture levels can also reduce LST by enabling more efficient heat transfer from the soil to the atmosphere through evaporation and transpiration. Mean annual rainfall is an important factor to consider when analyzing and interpreting LST data because it can significantly impact LST through its effects on vegetation cover, soil moisture, and evapotranspiration rate^35^.

NDVI can affect LST by regulating the amount of solar radiation absorbed by the land surface. A study by^36^ in China found a negative correlation between NDVI and LST, demonstrating that areas with more vegetation cover tend to have lower LST. Elevation is an important factor that affects LST because it influences the temperature of the air that comes into contact with the land surface. As elevation increases, the temperature of the air decreases, resulting in lower LST. Normalized Difference Built-up Index (NDBI) measures the amount and density of developed areas in an area. Built-up areas tend to have higher LST than non-built-up areas due to the heat generated by buildings and other infrastructure.

A study by^18^, in China found a positive correlation between NDBI and LST, showing that areas with higher NDBI tend to have higher LST. Population density can affect LST by altering the amount of built-up areas and vegetation cover in an area. A study by^18^ found that population density had a positive correlation with LST, indicating that more densely populated areas tend to have higher LST due to increased levels of development. Normalized difference water index (NDWI), which is a measure of water content in the environment, can also affect LST, with areas with higher water content generally having lower LST values^18^.

#### Ordinary least squares

The relationship between land surface temperature and several explanatory variables including air temperature, rainfall, NDBI, elevation, NDVI, and population density was investigated. The results indicate that air temperature has a positive and significant relationship with land surface temperature, with a coefficient of 0.02 (see Tables 1 and 2). This suggests that as air temperature increases, land surface temperature also increases. Similarly, rainfall has a positive and significant relationship with land surface temperature, with a coefficient of 0.062, indicating that increases in rainfall are associated with increases in land surface temperature*.*The NDBI coefficient, with a value of 9.332, shows a positive relationship with land surface temperature. This indicates that areas with high NDBI values, which may indicate an abundance of impervious surfaces, have higher land surface temperatures. However, the elevation coefficient is negative, with a coefficient of − 0.092, indicating that areas at higher elevations tend to have lower land surface temperatures. The NDVI coefficient is negative with a coefficient of − 7.8, indicating that areas with higher vegetation cover tend to have lower land surface temperatures. Lastly, population density has a positive and significant relationship with land surface temperatures, with a coefficient of 0.093. This suggests that areas with higher population density tend to have higher land surface temperatures.

**Table 1:** Calculated results of OLS (adjusted R-squared, Akaike’s information criterion and koenker statistic).

OLS performance indicators	Result
Adjusted R^2^	0.4155
Akaike’s Information Criterion	2162
Koenker (BP) Statistic	0.000

**Table 2:** Calculated result of OLS by intercepts, coefficients, standard error, and t-statistic.

Variable	Coefficient [a]	Standard error	t-statistic
Intercept	48.599773	1.925119	25.245073
Mean temperature	0.283	0.000087	3.251500
NDBI	9.322441	2.152419	4.331146
Elevation	− 0.9243	0.001156	− 7.996299
Population density	0.093	0.000015	6.204749
NDVI	− 7.808586	0.873421	− 8.940228
NDWI	− 1.514045	2.073181	− 0.730300

**Figure 5:**
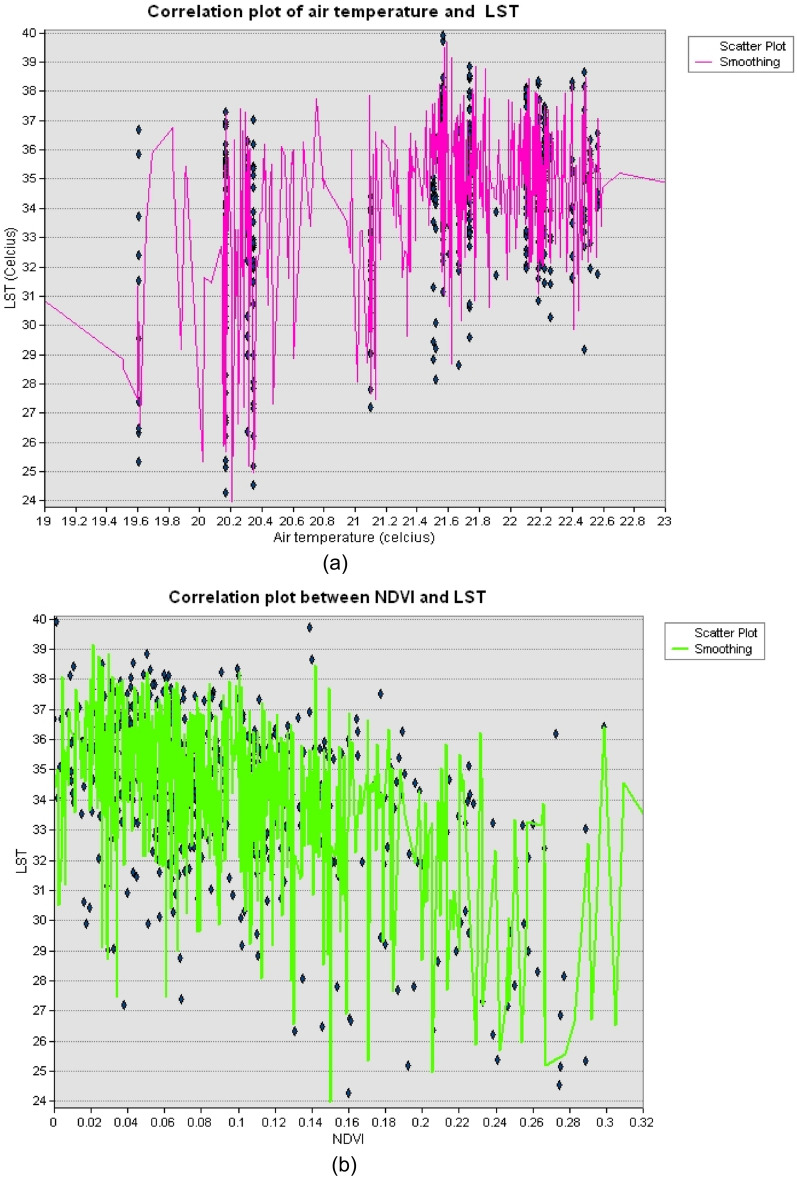

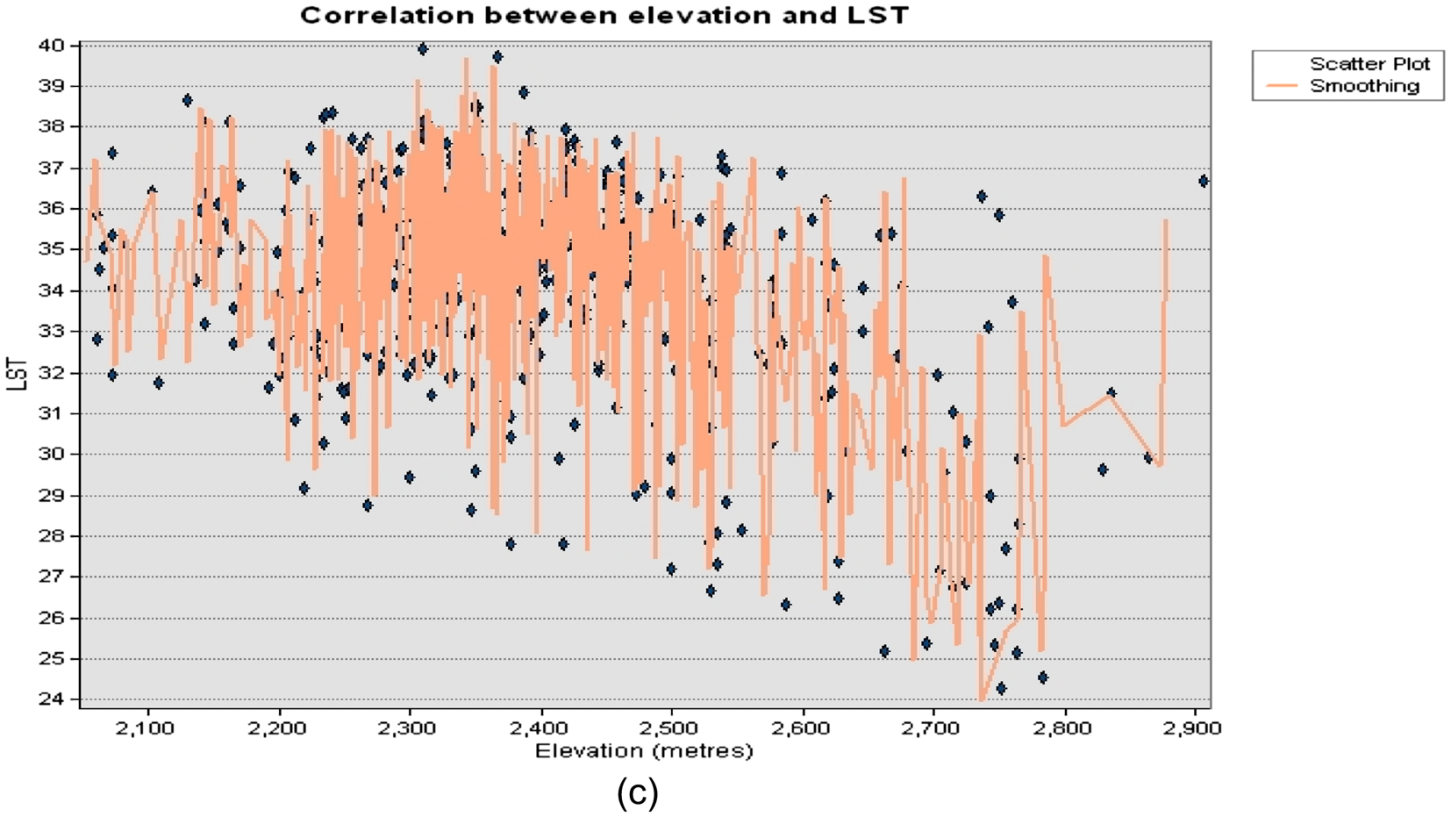
Correlation plot between selected explanatory variables and LST.

The results indicates that air temperature, rainfall, NDBI, elevation, NDVI, and population density are all important explanatory variables for land surface temperature. Lastly, the NDWI coefficient has a negative value of − 1.51, indicating that areas with higher water content tend to have lower land surface temperatures. This aligns with previous research that has highlighted the cooling effect of water on land surface temperature. The results are visually depicted in Fig. 5a–c using scatterplots. The findings have important implications for land and urban planning and can inform efforts to mitigate and adapt to the impact of urbanization and climate change on land surface temperature.

#### Non-stationarity test

The Koenker Statistic is an extension of the Breusch-Pagan test, which was developed to detect heteroscedasticity in regression models^37^. In empirical studies, it is often essential to understand the strength and reliability of the relationship between independent variables and the dependent variable. The Koenker Statistic provides a valuable tool for researchers to evaluate the consistency of this relationship, enabling them to make informed decisions about the validity of their models. This makes it particularly useful in analyzing spatial data and models that exhibit variations across different regions or data points^18^.

As the p-value of the Koenker's studentized Breusch-Pagan statistic is 0.000, it indicates strong evidence to reject the null hypothesis of homoscedasticity in the residuals of the OLS regression model^37^. As the residuals of the OLS regression model are heteroscedastic, the model is also non-stationary. Non-stationarity occurs when statistical properties of a process change over time. Hence, it is essential to account for the non-stationarity in the data, such as by using alternative models such as Weighted Least Squares (WLS) or Geographically Weighted Regression (GWR), which take into account local variations in the data^18^.

#### Geographically weighted regression

Geographic weighted regression (GWR) is a powerful spatial analysis technique used to model spatially varying relationships between a dependent variable and multiple explanatory variables^38^. In this case, the dependent variable is land surface temperature (LST), and the explanatory variables are temperature, normalized difference built-up Index (NDBI), elevation, population density, normalized difference vegetation Index (NDVI), and normalized difference water Index (NDWI).

**Figure 6:**
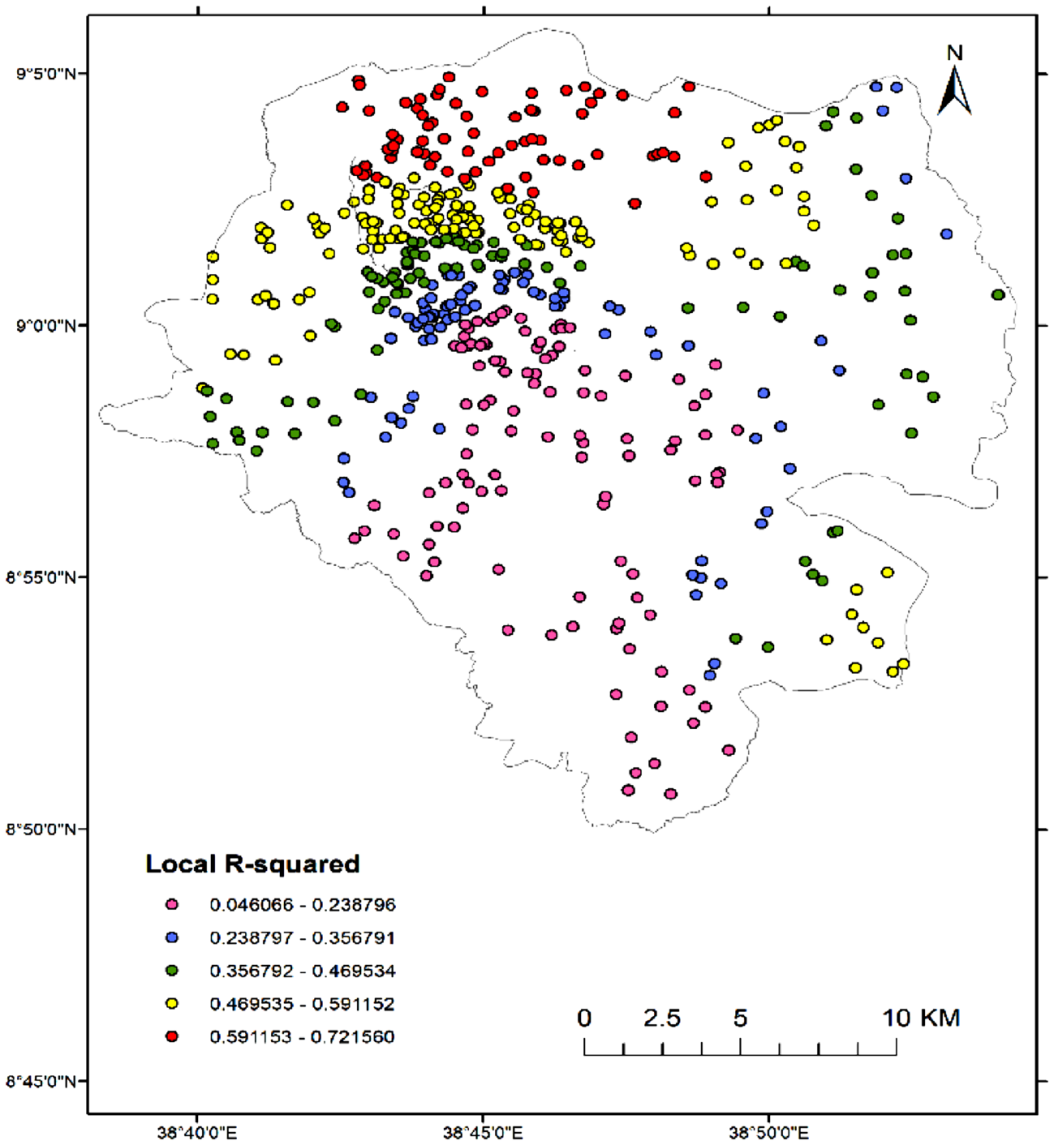
Map of local R-squared result of GWR. (Map created by author using QGIS 3.22 software: https://qgis.org/).

The standardized residuals of the GWR model represent the differences between the predicted and observed values of the dependent variable, adjusted for the variance explained by the independent variables. The result suggests that the standardized residuals of the GWR model are distributed between − 3.60 and 2.91 standard deviations from the mean (see Fig. 6). A residual that is larger than three times the standard deviation from the mean is often considered an outlier. Thus, having standardized residuals between − 3.60 to 2.91 implies that the GWR model may have some outliers, but the range of standardized residuals is still within a reasonable range.

#### Model performance comparison using coefficients and AIC

There are several techniques that can be used to compare the performance between OLS and GWR models (Mccann et al.^38^, Zhao et al.^18^):

1. Adjusted R-squared (coefficient of determination) is a commonly used measure of goodness of fit in regression models. Its value varies from 0.0 to 1.0, with higher values being preferable. It may be interpreted as the proportion of dependent variable variance accounted for by the regression model^39^.
2. Comparison of AICc values: The Akaike information criterion (AIC) is the second tool to compare the performance of OLS models and GWR models (Akaike^41^). AIC evaluates the goodness of fit of the model while penalizing it for its complexity. The model with the lowest AIC value is preferred because it provides the best trade-off between goodness of fit and complexity. If the AICc values for two models differ by more than 3, the model with the lower AICc is held to be better (Luo and Peng^40^).
3. Standardized residual analysis: The third comparison mechanisms are standardized residuals. Standardized residuals are calculated by dividing the residuals by their estimated standard deviation. Standardized residuals follow a standard normal distribution with a mean of zero and a standard deviation of one. The range of values for standardized residuals should be between three and + 3 for a well-fitted model (Feng et al.^42^).

The difference in R-squared values between OLS and GWR in the study suggests that spatial heterogeneity in the relationship between land surface temperature and its explanatory variables could exist. In previous studies, the use of GWR has been found to improve the accuracy of spatial models and provide insights into spatial variations that cannot be obtained using traditional OLS models. A study by^40^ compared the performance of OLS and GWR in modeling the relationships between land surface temperature and urbanization factors in Hangzhou, China. The study found that GWR improved the accuracy of the model by capturing the spatial variations in the relationships between the variables.

As it is presented in Table 3, the OLS model has an adjusted R squared value of 0.4155 and AIC value of 2162, while the GWR model has an adjusted R squared value of 0.577 and AIC value of 1052.188. The adjusted R squared takes into account the number of independent variables in the model and penalizes the R squared value for the addition of unnecessary variables, so a higher adjusted R squared value indicates a better fit of the model. In terms of adjusted R squared, the GWR model performs better than the OLS model because of the higher value. An adjusted R squared of 0.577 indicates that the GWR model explains approximately 57.7% of the variance in the response variable, which is greater than the OLS model. The adjusted R squared value accounts for the number of predictor variables in the model, so a higher adjusted R squared indicates that the model is less likely to over fit compared to the model with a lower value.

**Table 3:** Comparision between OLS and GWR using adjusted R-squared,AIC and StdResiduals.

Model	Adjusted R^2^	AIC	Standardized residual
OLS	0.42	2162.0	− 3.66 to + 3.66
GWR	0.57	1052.1	− 3.60 to + 2.91

The AIC is used to compare models based on their goodness of fit and complexity, where a lower AIC value indicates a better-performing model^41^.This is likely because GWR is better equipped to handle spatial heterogeneity in the data and can model relationships that vary based on location, whereas OLS assumes a constant relationship across all points. In terms of AIC, a lower value indicates a better fit for the model^41^. The GWR model has a lower AIC value than the OLS model, which indicates better performance by the GWR model. Therefore, in this case, the GWR model outperforms the OLS model based on both adjusted R squared and AIC values^40^.

Standardized residuals are a commonly used metric to assess the goodness-of-fit of regression models^42^. A standardized residual is the residual divided by the estimated standard deviation of the residual, and it represents the distance of an observation from the fitted regression line in units of standard deviation. The standardized residuals range for the OLS model is between − 3.66 and + 3.66, meaning it has some outlier values that don’t correspond to a normal distribution. The GWR model has a standardized residuals range between − 3.60 and + 2.91, which suggests that the model is handling outliers better and providing predictions that are more reliable. Therefore, based on the range of standardized residuals, we can infer that the GWR model performs better than the OLS model.

## Discussion

### Land surface temperature and explanatory variables

The study aimed to investigate the relationship between land surface temperature (LST) and several explanatory variables, including air temperature, Normalized Difference Vegetation Index (NDVI), elevation, Normalized Difference Built-up Index (NDBI), population density, and mean annual rainfall. The findings revealed significant relationships between LST and the examined explanatory variables, which contribute to our understanding of the spatial distribution of surface temperature and its drivers. Several studies have reported a significant positive correlation between LST and air temperature. A positive correlation between LST and air temperature is a common finding in many studies, suggesting that air temperature is an important factor influencing LST. For example, a study in India^43^ found a strong positive correlation between LST and air temperature in urban areas, while a study in the United States found a positive correlation between LST and air temperature in suburban areas*.* This finding is consistent with previous research that highlights the influence of air temperature on surface temperature patterns. Higher air temperatures contribute to increase LST, leading to the formation of urban heat islands and potential adverse effects on human health and energy consumption.

Similar studies revealed that factors such as land use and urbanization play a crucial role in amplifying the impact of air temperature on land surface temperature (LST). For instance, a study conducted in China demonstrated a stronger correlation between air temperature and LST in areas with high levels of urbanization. Meanwhile, a study in Brazil found that urban density exhibited a more significant relationship with LST compared to factors like vegetation cover or temperature^44,45^. The positive relationship between LST and air temperature is well-established in the literature, and previous studies have consistently found air temperature to be a significant predictor of LST in urban areas. Another study^45^ investigated the relationship between LST and air temperature in a mountainous region in China. They found that there was a positive correlation between LST and air temperature, and the strength of the correlation varied depending on the altitude. Specifically, the correlation was stronger at lower altitudes and weaker at higher altitudes.

The negative coefficient between land surface temperature and NDVI in the study implies that as NDVI increases, land surface temperature decreases. A study by^46^ found that NDVI has a negative correlation with LST, indicating the importance of increasing vegetation cover to reduce LST. The negative relationship between LST and elevation has been reported in several previous studies. For example, a study^47^ found that LST decreased with increasing elevation. The positive coefficient between LST and normalized difference built-up index (NDBI) in the study suggests that as the NDBI increases, the LST also increases. This is in line with previous research that has found a positive correlation between NDBI and LST. For example, a study in Istanbul, Turkey^48^ also found a positive correlation between NDBI and LST, with higher NDBI values indicating a higher proportion of impervious surfaces and resulting in higher LST values.

The positive coefficient between land surface temperature and population density in the study suggests that as population density increases, so does land surface temperature. This relationship has been observed in various previous studies. Population density has been found to have a positive relationship with LST, with several studies reporting a higher LST in areas with a higher population density. For example, a study conducted in Japan^49^ found a positive correlation between land surface temperature and population density, suggesting that urbanization and population growth contribute to the urban heat island effect. Similarly, a study in Shenzhen^50^ also found a positive relationship between population density and land surface temperature, which was attributed to the effects of urbanization and anthropogenic heat .However, it's worth noting that not all studies have found a positive relationship between land surface temperature and population density. This study has found a positive correlation between land surface temperature (LST) and mean annual rainfall. Several previous studies have examined the relationship between LST and rainfall, A study by^51^ found a negative correlation between LST and rainfall in the urban areas of Beijing, China.

### Performance difference between the two models

The results of the ordinary least squares (OLS) and geographically weighted regression (GWR) analyses indicate that the land surface temperature (LST) is moderately correlated with air temperature, NDVI, elevation, NDBI, population density, and mean annual rainfall, but the strength of the correlation may vary depending on geographic location. The OLS R-squared value of 0.42 indicates that these variables can explain 42% of the variance in LST across the entire study area. On the other hand, the higher GWR R-squared of 0.57 indicates a stronger and more spatially varying relationship between these variables and LST.

This finding is consistent with previous studies that have demonstrated that the relationship between LST and its predictors is often complex and spatially varying. A study by^52^ found a strong linear relationship between mean surface temperature and the percentage of impervious areas in urban areas, while other studies have found that the relationship between LST and vegetation cover is nonlinear and varies depending on the type of vegetation.

The results of the ordinary least squares (OLS) and geographically weighted regression (GWR) revealed the GWR model outperformed the ordinary least squares (OLS) model in terms of the Akaike Information Criterion (AIC) value. Several studies have previously shown the advantages of using GWR over OLS in spatial analyses. Studies by^53–55^ demonstrated the superiority of GWR over OLS in modeling spatially varying coefficients. In the present study, the standardized residuals for OLS range from − 3.65 to + 3.56, while the standardized residuals for GWR range from − 3.59 to + 2.91. The fact that both models have standardized residuals within the range of − 3 to + 3 suggests that they are both acceptable in terms of fit. The comparison of standardized residuals between OLS and GWR in the present study suggests that both models have acceptable fit.

Another study by^56^ used both OLS and GWR models to investigate the relationship between LST and environmental factors such as land use, population density, and elevation in Beijing, China. The study found that the GWR model had better performance in terms of model fit and spatial autocorrelation compared to the OLS model. Similarly, a study by^57^ utilized both OLS and GWR regression models to study the relationship between LST and the study found that the GWR model had a better fitting performance than the OLS model. Another study^58^ compared the performance of OLS and GWR models in studying the LST-LULC relationships and the result demonstrate that the GWR model has several advantages over the OLS model in studying the relationship between LST and environmental factors, particularly in capturing the spatial heterogeneity of LST.

## Conclusion

The research aimed to compare the performance of Least Squares Regression (LSR) and Geographically Weighted Regression (GWR) in modeling land surface temperature (LST) in Addis Ababa city, Ethiopia. Through the analysis and evaluation, the researcher have gained valuable insights into the strengths and limitations of each method. The findings indicate that both LSR and GWR are effective in modeling LST in Addis Ababa city. LSR provides a global perspective and produces a single model that represents the entire study area. A simple and computationally efficient method provides reliable results when spatial variability is relatively low. On the other hand, GWR takes into account the spatial non-stationarity of LST and produces localized models that capture the spatial variations within the study area. GWR is particularly useful when there are significant spatial variations in LST across the city.

Additionally, GWR allows for the identification of spatially varying relationships between LST and the predictor variables, which can provide valuable insights into the underlying drivers of LST variations. This localized analysis can help inform policy and decision-making processes related to urban planning, climate change mitigation, and public health interventions. Moreover, the author plans to engage in discussions and conduct workshops to promote a more profound comprehension of the study findings and stimulate conversations regarding potential strategies for adaptation and measures for mitigation. Based on the findings of the research, the researcher offer the following recommendations for future studies and applications(a)it is recommended to consider the spatial non-stationarity of LST when modeling and analyzing urban temperature patterns. GWR provides a powerful tool for capturing and understanding the localized variations in LST, which can assist in formulating targeted interventions and policies. (b)Longitudinal studies are recommended to investigate the temporal dynamics of LST and its relationship with other environmental and socio-economic factors. Understanding the temporal variations in LST can aid in predicting future temperature changes and designing effective climate adaptation strategies. By considering the strengths and limitations of both LSR and GWR, researchers and policymakers can make informed decisions and develop targeted interventions to mitigate the adverse impacts of urban heat islands.

## Data Availability

The land surface temperature (LST), normalized difference vegetation index (NDVI), normalized difference built-up index (NDBI), and normalized difference water index (NDWI) datasets can be accessed from the website https://lpdaac.usgs.gov. The dataset containing information on elevation is also available at the same website. For the climatology of air temperature and rainfall, the dataset can be found at https://www.worldclim.org. Lastly, the dataset on population density can be obtained from https://www.worldpop.org.
